# Disparities in patient share and characteristics between disease-modifying therapy-treated and -untreated people with multiple sclerosis in Germany: a claims data analysis from 2017 to 2022

**DOI:** 10.3389/fneur.2025.1561810

**Published:** 2025-04-15

**Authors:** Ann-Sophie Stratil, Steffeni Papukchieva, Nils-Henning Neß, Inge Wolf, Antonios Bayas, Peter Flachenecker, Benjamin Friedrich

**Affiliations:** ^1^Temedica GmbH, Munich, Germany; ^2^Hexal AG, Holzkirchen, Germany; ^3^Department of Neurology, Faculty of Medicine, University of Augsburg, Augsburg, Germany; ^4^Neurological Rehabilitation Center Quellenhof, Bad Wildbad, Germany

**Keywords:** multiple sclerosis, real-world data, healthcare utilization, disease modifying therapy-untreated, regionality

## Abstract

**Introduction:**

Despite significant advances in multiple sclerosis (MS) treatment, a substantial proportion of people with MS (pwMS) remains untreated with disease-modifying therapies (DMTs). This study aimed to assess the proportion of DMT-untreated pwMS according to clinical and sociodemographic characteristics, the differences in healthcare utilization patterns, including MRI frequency and physiotherapy use, between DMT-treated and DMT-untreated pwMS, as well as to examine the time to first prescription among newly diagnosed pwMS in Germany from 2017 to 2022.

**Methods:**

Longitudinal routine data from 4.5 million individuals insured by German statutory health insurance were analyzed. PwMS were identified by ICD-10-GM code G35 in at least two quarters within a calendar year. PwMS who filled a DMT prescription during the observation period were classified as DMT-treated. Newly diagnosed pwMS had no recorded G35 code and DMT prescription in the 2 years prior to initial MS coding. MRI scans and physiotherapy sessions were identified using OPS codes and the German *Heilmittelkatalog*. Group differences were tested with two-sided χ^2^-tests and *t*-tests.

**Results:**

In total, 45.6% of pwMS were DMT-untreated. DMT-untreated rates were higher in secondary progressive (SPMS) and primary progressive MS (PPMS) than relapsing-remitting MS (RRMS; *p* < 0.0001). Older pwMS (>45 years) were more frequently DMT-untreated (56.0%) compared to younger pwMS (≤45 years; 27.4%; *p* < 0.0001). Regional differences ranged from 37.5% in eastern Germany to 54.0% in the south-west. More than half of newly diagnosed pwMS did not receive their first prescription for DMT within 6 months of their initial MS coding, with longer times to first DMT prescription among pwMS with a progressive compared to a relapsing disease onset (*p* < 0.0001). DMT-untreated pwMS averaged more physiotherapy sessions (DMT-untreated: 23.7, SD 35.0; DMT-treated: 20.2, SD 28.7; *p* < 0.0001) and less MRI scans (DMT-untreated: 0.8, SD 0.8; DMT-treated: 1.5, SD 0.8; *p* < 0.0001) annually over the observation period than DMT-treated pwMS.

**Conclusion:**

These real-world data offer valuable insights into patient care and treatment allocation.

## 1 Introduction

In 2023, an estimated 280,000 individuals in Germany were affected by multiple sclerosis (MS), a chronic inflammatory and neurodegenerative disorder of the central nervous system characterized by demyelination, axonal damage, and neurodegeneration (1, 2). MS is classified into three phenotypes: relapsing-remitting multiple sclerosis (RRMS), marked by episodes of relapse followed by periods of recovery; secondary progressive multiple sclerosis (SPMS), which involves a progressive accumulation of disability after an initial relapsing-remitting phase; and primary progressive multiple sclerosis (PPMS), characterized by a steady accumulation of disability from onset without distinct remissions (3). At onset, RRMS is the most prevalent disease course, affecting ~85% of people with MS (pwMS), followed by PPMS, which accounts for about 15% of cases. Approximately 65% of pwRRMS eventually transition to SPMS (2).

Major advances in the therapeutic landscape for MS in the last 25 years have revealed effective disease-modifying therapies (DMTs) characterized by an acceptable safety profile with significant impact on disease progression, reduced disability accumulation and patients' quality of life (4–6). The choice of DMT depends on the severity and frequency of relapses, MRI activity, and evidence of disease progression (1). In Germany, only 30%−60% of pwMS receive DMTs regardless of being eligible for treatment according to clinical guidelines (7–10). PwRRMS are more likely to receive DMTs than those with SPMS or PPMS. Despite therapeutic advances, progressive MS remains a challenge, with limited DMT options (1, 6).

Magnetic resonance imaging (MRI) is essential for diagnosis, prognosis, and monitoring of disease progression and treatment effectiveness in MS (11). Regular MRI scans for pwMS at 6 to 12 month intervals are recommended (12, 13). Only 84.8% of German pwRRMS with highly active disease received at least one MRI in 2016 (14), while figures for people with other MS types have not been reported. Symptomatic treatment, such as physiotherapy, is another important cornerstone of MS management, aiming to improve or restore functional abilities (1, 15, 16). Certain MS subtypes appear to rely more heavily on physiotherapy than others; for example, a claims data analysis from Germany (2011–2015) found that pwSPMS and pwPPMS use physiotherapy more frequently than pwRRMS (17). Given the challenges of managing progressive MS with a limited number of DMTs approved, it may be speculated that DMT-untreated pwMS with higher disability may more frequently use symptomatic therapies, such as physiotherapy, but receive less monitoring of disease activity through MRI scans.

This study aimed to assess the proportion of DMT-untreated pwMS according to clinical and sociodemographic characteristics, the differences in healthcare utilization patterns, including MRI frequency and physiotherapy use, between DMT-treated and DMT-untreated pwMS, as well as to examine the time to first prescription among newly diagnosed pwMS in Germany from 2017 to 2022. Analyses were based on real-world claims data from 4.5 million individuals insured by German statutory health insurance (SHI).

## 2 Materials and methods

### 2.1 Ethics approval and consent to participate

This study did not require ethical board approval or written informed patient consent since the data analyzed retrospectively was anonymized claims data.

### 2.2 Dataset and data processing

This analysis used longitudinal routine data from German SHIs, encompassing both corporate health insurers and regular insurers. The dataset covers ~4.5 million insured individuals. The data consists of anonymized and aggregated patient-level information, including disease diagnoses based on the International Statistical Classification of Diseases and Related Health Problems, 10th revision, German modification (ICD-10-GM), Version 23 codes, sociodemographic characteristics, as well as prescriptions for treatments and procedures. The data were stored in the Permea platform (Temedica GmbH) in a General Data Protection Regulation (GDPR) compliant manner, and no personal information, that might allow identification of individuals, was revealed. Data for specific subgroups were available only if the subgroup comprised a minimum of five individuals.

### 2.3 Cohort definitions

The total cohort comprised pwMS, defined as individuals who received an ICD-10-GM code G35.0 (initial manifestation of MS), G35.1- (RRMS), G35.2- (PPMS), G35.3- (SPMS), and/or G35.9 (unspecified MS) in at least two quarters of the same calendar year between January 2017 and December 2022.

PwMS were considered DMT-treated if they filled a prescription for at least one of the following DMTs at any point during the observation period or the respective calendar year, depending on the level of analysis, identified by the corresponding Anatomical Therapeutic Chemical (ATC) code: dimethyl fumarate, diroximel fumarate, glatiramer acetate, interferon beta-1a, interferon beta-1b, peginterferon beta-1a, teriflunomide, cladribine, fingolimod, ozanimod, siponimod, ponesimod, alemtuzumab, natalizumab, ocrelizumab, ofatumumab, and rituximab [off-label].

PwMS who had no recorded G35 code and DMT prescription in the 2 years prior to their initial MS coding were classified as newly diagnosed. The initial MS coding was used to determine the type of disease onset as either relapsing (G35.1), progressive (G35.2 and G35.3), or unspecified (G35.0 and G35.9).

PwMS meeting the inclusion criteria could contribute to each MS type and, in the case of DMT-treated pwMS, DMT cohort only once during the whole observation period and each calendar year. However, they could contribute to multiple different cohorts in cases of different MS subtype codings (e.g., progression to SPMS or miscodings) and if they received several different DMTs.

### 2.4 Variables and statistical methods

To assess the proportion of DMT-untreated pwMS according to clinical and sociodemographic characteristics, and the differences in healthcare utilization patterns between DMT-treated and DMT-untreated pwMS, sociodemographic characteristics, including age group, sex, and the 1-digit postal code region of the patient's residence at the most recent patient record, were analyzed using absolute and relative frequencies across the entire observation period for both DMT-treated and -untreated pwMS separately. Age groups were categorized as 0–17, 18–25, 26–35, 36–45, 46–55, 56–65, 66–75, and 76+, or as ≤ 45 years and >45 years. The 45-year threshold was chosen based on clinically relevant factors, such as differences in disease progression, treatment initiation, and management strategies that typically occur around this age. For the analysis of regional distribution, estimated pwMS numbers were adjusted based on age and sex to align with the demographic patterns of each region, ensuring accurate representation of the population structure. The average number of physiotherapy sessions per pwMS was based on the *German Heilmittelkatalog* coding (remedies listed under therapeutic area of physiotherapeutic interventions and diagnostic groups of disorders of the musculoskeletal system, disorders of the nervous system, disorders of the internal organs and other disorders). The average number of MRIs per pwMS was based on the German *Operationen- und Prozedurenschlüssel* codes (OPS codes 3-800, 3-802, 3-820, 3-823). While this study is primarily descriptive and does not aim to make causal inferences, we conducted statistical tests for group differences to provide a context for the observed patterns. These tests assess whether the observed sub-group differences are statistically significant within the scope of the descriptive analysis. However, these tests do not account for potential confounding variables, and as such, *p*-values should not be interpreted as evidence of causal relationships. Group differences were analyzed using two-sided χ^2^-tests for categorical variables and *t*-tests for continuous variables. To account for multiple comparisons, Bonferroni corrections were applied, with adjusted significance thresholds varying based on the number of comparisons performed in each analysis. Among the total cohort of pwMS, within each clinically distinct MS subtype, comparisons were made between DMT-treated and untreated groups. Additionally, cross-subtype comparisons were made between DMT-treated and DMT-untreated pwMS, using RRMS as the reference group. Within subtype and cross-subtype comparisons were restricted to clinically distinct MS subtypes (RRMS, SPMS, and PPMS).

To examine the time to first prescription among newly diagnosed pwMS, time from diagnosis to the first prescription of a DMT were assessed using absolute and relative frequencies. Time to first prescription was grouped as 0–6 months after initial MS coding, >6 months after initial MS coding and DMT-untreated (i.e., newly diagnosed pwMS who did not receive a DMT prescription within the observation period). Newly diagnosed pwMS who received their first DMT prescription within 6 months, more than 6 months, or remained DMT-untreated were compared based on relapsing vs. progressive onset type using two-sided χ^2^-tests. Comparisons across all MS types were also made by age and gender, with Bonferroni correction applied to account for multiple comparisons.

Data processing and statistical tests were performed using Python 3.9. Figures were generated using GraphPad Prism 10.

## 3 Results

### 3.1 Patient population

The dataset comprised 13,214 pwMS from 2017 to 2022 of which 2,687 were newly diagnosed. RRMS coding was most prevalent (73.1%), followed by SPMS coding (23.6%) and PPMS coding (29.1%; Table 1). This includes pwMS who received coding for several MS types (e.g., RRMS and SPMS) during the observation period. 66.6% of the total cohort were female, and 63.6% were >45 years. Among newly diagnosed pwMS, 49.6% had a relapsing, 2.9% a progressive, and 47.5% had an unspecified disease onset. 64.4% of newly diagnosed pwMS were female, and 44.5% were >45 years.

**Table 1:** Patient populations.

**MS type**	**Number of pwMS (%)**	**Sex**		**Age**	
		**Female (%)**	**Male (%)**	≤ **45 years (%)**	>**45 years (%)**
All types	13,214 (100%)	8,803 (66.6%)	4,411 (33.4%)	4,806 (36.4%)	8,408 (63.6%)
Initial manifestation of MS	3,981 (30.1%)	2,707 (68%)	1,274 (32%)	1,759 (44.2%)	2,222 (55.8%)
RRMS	9,654 (73.1%)	6,547 (67.8%)	3,107 (32.2%)	3,993 (41.4%)	5,661 (58.6%)
PPMS	3,839 (29.1%)	2,497 (65%)	1,342 (35%)	940 (24.5%)	2,899 (75.5%)
SPMS	3,124 (23.6%)	2,040 (65.3%)	1,084 (34.7%)	548 (17.5%)	2,576 (82.5%)
Unspecified MS	12,522 (94.8%)	8,383 (66.9%)	4,139 (33.1%)	4,546 (36.3%)	7,976 (63.7%)

Characteristics of pwMS by MS type 2017-2022. Characteristics are based on the total cohort of pwMS, defined as individuals who received an ICD-10-GM code for MS (Initial manifestation of MS, G35.0; RRMS, G35.1-; PPMS, G35.2-; SPMS, G35.3-; and/or Unspecified MS, G35.9) in at least two quarters in the same calendar year between January 2017 and December 2022. MS, multiple sclerosis; RRMS, relapsing-remitting MS; SPMS, secondary progressive MS; PPMS, primary progressive MS; pwMS, people with MS.

### 3.2 DMT-untreated population

**Figure 1:**
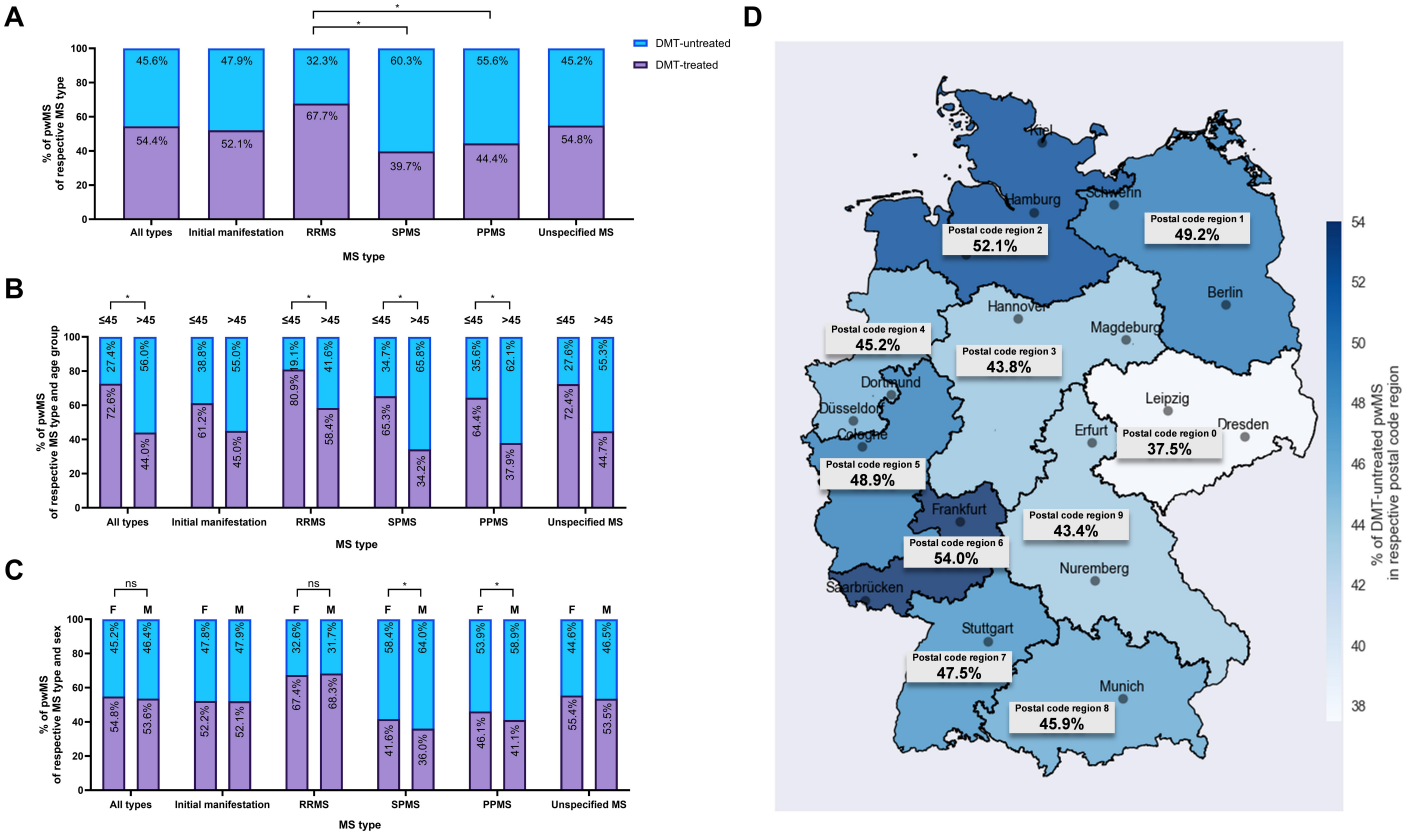
Characteristics of DMT-treated and -untreated pwMS (2017–2022). **(A)** Proportion of DMT-treated and -untreated pwMS, categorized by MS type. **(B)** Proportion of DMT-treated and -untreated pwMS among individuals categorized by MS type and age group. **(C)** Proportion of DMT-treated and -untreated pwMS categorized by MS type and sex. **(D)** Proportion of DMT-untreated pwMS by the 1-digit postal code region of residence. This analysis includes the total cohort of pwMS, defined as individuals who received an ICD-10-GM code for MS (Initial manifestation of MS, G35.0; RRMS, G35.1-; PPMS, G35.2-; SPMS, G35.3-; and/or Unspecified MS, G35.9) in at least two quarters in the same calendar year between January 2017 and December 2022. DMT, disease-modifying therapy; MS, multiple sclerosis; RRMS, relapsing-remitting MS; SPMS, secondary progressive MS; PPMS, primary progressive MS; pwMS, people with MS. Differences in proportions of untreated pwMS between subgroups were assessed using χ^2^-tests, with significance levels adjusted for multiple comparisons using the Bonferroni correction (ns, not significant; *significant after Bonferroni correction).

Of the total cohort of 13,214 pwMS between 2017 and 2022, 45.6% were not treated with DMTs (Figure 1A). The proportion of DMT-untreated pwMS was significantly higher in pwSPMS (60.3%) and pwPPMS (55.6%) compared to those with RRMS (32.3%; *p* < 0.0001, respectively). Between 2017 and 2022, the proportion of DMT-untreated pwRRMS remained stable (Supplementary Figure 1). The proportion of DMT-untreated pwSPMS and pwPPMS decreased significantly over the observation: from to 72.8% to 67.2% (*p* < 0.001), and from 65.9% to 61.5% (*p* < 0.01), respectively.

Older pwMS (>45 years) were more frequently DMT-untreated compared to younger pwMS ( ≤ 45 years; *p* < 0.0001; Figure 1B). This age-related difference was consistent within RRMS, SPMS and PPMS (*p* < 0.0001 for each MS subtype).

In pwRRMS, rates of being DMT-untreated were similar between females and males (Figure 1C). In pwSPMS and pwPPMS, males were more frequently DMT-untreated than females (*p* < 0.01, respectively).

Geographic disparities were observed in DMT-untreated rates, with the highest rate in postal code region 6, located in south-western Germany, and the lowest in postal code region 0, located in eastern Germany (Figure 1D).

### 3.3 Time to first prescription

**Figure 2:**
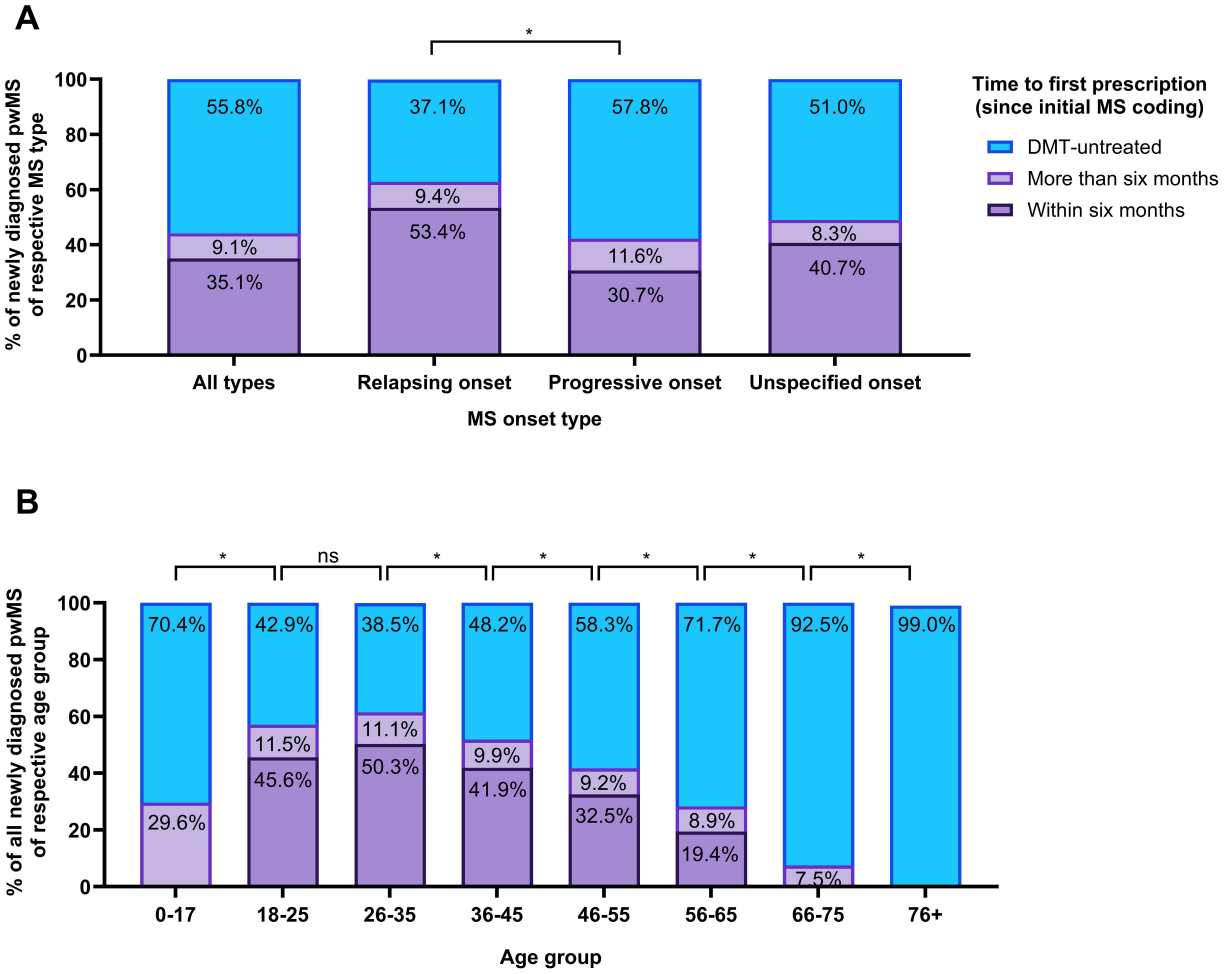
Time to first prescription since initial MS coding among newly diagnosed pwMS (2017–2022). **(A)** Proportion of newly diagnosed pwMS, categorized by MS onset type, who filled a first prescription for a relevant DMT within 6 months or after more than 6 months since their initial MS coding or who remained DMT untreated throughout the observation period. **(B)** Proportion of newly diagnosed pwMS of all onset types, categorized by age group who filled a first prescription for a relevant DMT within 6 months or after more than 6 months since their initial MS coding or who remained DMT-untreated throughout the observation period. This analysis includes newly diagnosed pwMS defined as individuals with no recorded G35 code and DMT prescription in the 2 years prior to their initial MS coding. DMT, disease-modifying therapy; MS, multiple sclerosis; pwMS, people with MS. Differences in proportions of pwMS who received their first DMT prescription within 6 months vs. more than 6 months or remained DMT-untreated were assessed using χ^2^-tests, with significance levels adjusted for multiple comparisons using the Bonferroni correction (ns, not significant; *significant after Bonferroni correction).

64.9% of all newly diagnosed pwMS did not receive their first DMT within 6 months of their initial MS coding: 9.1% received their first DMT more than 6 months after their initial MS coding, and 55.8% remained DMT-untreated between 2017 and 2022 (Figure 2A). The share of pwMS not receiving a first prescription for a DMT within 6 months of their initial MS coding was higher among pwMS with a progressive compared to a relapsing disease onset (*p* < 0.0001).

The proportion of newly diagnosed pwMS not receiving a first DMT within 6 months of their initial MS coding varied by age (Figure 2B). From age 26, the proportion of pwMS not receiving a first DMT within 6 months of initial MS coding increased with each successive age group (*p* < 0.01, respectively). The highest shares of newly diagnosed pwMS who did not receive any DMTs in the observation period were observed in the youngest (0–17 years), and oldest (66–75 and 76+ years) age groups, with 70.4%, 92.5%, and 99.0%, respectively. Analysis of age group distribution within relapsing vs. progressive onset type was not feasible due to insufficient sample sizes.

Between female and male pwMS, the percentage of newly diagnosed pwMS not receiving a first prescription for a DMT within 6 months of their initial MS coding was similar (Supplementary Figure 2).

### 3.4 Healthcare utilization

**Figure 3:**
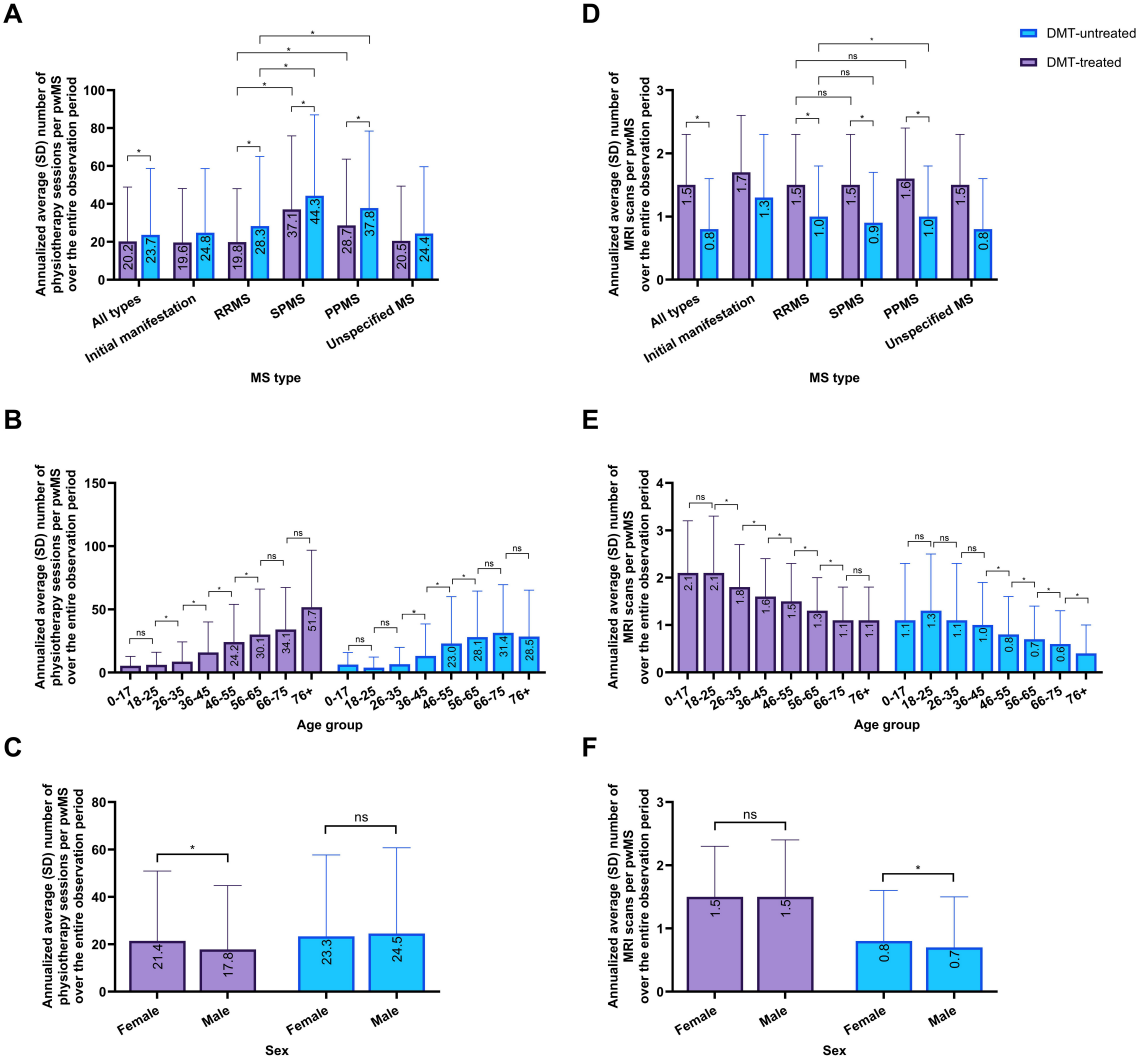
Healthcare resource utilization patterns of DMT-treated and -untreated pwMS (2017–2022). **(A–C)** Annualized average number (SD) of physiotherapy sessions per pwMS among DMT-treated and -untreated pwMS over the observation period**. (A)** Categorized by MS type. **(B)** Categorized by age group for all MS types. **(C)** Categorized by sex for all MS types. **(D–F)** Annualized average number (SD) of MRI scans per pwMS among DMT-treated and untreated pwMS over the observation period. **(D)** Categorized by MS type. **(E)** Categorized by age group for all MS types. **(F)** Categorized by sex for all MS types. This analysis includes the total cohort of pwMS, defined as individuals who received an ICD-10-GM code for MS (Initial manifestation of MS, G35.0; RRMS, G35.1-; PPMS, G35.2-; SPMS, G35.3-; and/or Unspecified MS, G35.9) in at least two quarters in the same calendar year between January 2017 and December 2022. DMT, disease-modifying therapy; MRI, magnetic resonance imaging; MS, multiple sclerosis; RRMS, relapsing-remitting MS; SD, standard deviation; SPMS, secondary progressive MS; PPMS, primary progressive MS; pwMS, people with MS. Differences between subgroups were assessed using *t*-tests, with significance levels adjusted for multiple comparisons using the Bonferroni correction (ns, not significant; *significant after Bonferroni correction).

In the total cohort of pwMS, DMT-untreated pwMS received more annual physiotherapy sessions per pwMS (23.7, standard deviation SD 35.0) over the complete observation period than DMT-treated pwMS (20.2, SD 28.7; *p* < 0.0001; Figure 3A). This pattern was true across MS subtypes (p < 0.0001 for each MS subtype). Additionally, pwSPMS and pwPPMS received more physiotherapy sessions than pwRRMS, regardless of treatment status (*p* < 0.0001, respectively).

A trend was observed where older pwMS received more annual physiotherapy sessions on average than younger pwMS, irrespective of DMT treatment status (Figure 3B). Among DMT-treated individuals, annual physiotherapy sessions increased significantly with each successive age group from age 18 to 65 (*p* < 0.001, respectively). A similar trend was observed in DMT-untreated pwMS, where annual physiotherapy sessions increased significantly with each successive age group from age 36 to 65 (*p* < 0.001, respectively). This age-related trend in physiotherapy utilization was consistent across MS subtypes (Supplementary Figure 3A). Among DMT-treated pwMS, females received more physiotherapy sessions per year than males (*p* < 0.0001; Figure 3C). A similar pattern was observed across all MS types (Supplementary Figure 3C).

Conversely, DMT-untreated pwMS received fewer annual MRI scans (0.8, SD 0.8) per pwMS over the complete observation period than DMT-treated pwMS (1.5, SD 0.8; Figure 3D; *p* < 0.0001). This pattern was observed across MS subtypes (*p* < 0.0001 for each MS subtype). Among DMT-untreated pwMS, pwSPMS received less MRI scans than pwRRMS (*p* < 0.0001).

An age-related trend in MRI utilization was also observed, with younger pwMS receiving more MRI scans than older individuals, regardless of treatment status (Figure 3E). Among DMT-treated individuals, annual MRI scans decreased significantly with each successive age group from age 18 to 75 (*p* < 0.001, respectively). Among DMT-untreated pwMS, MRI scan frequency declined significantly from age 36 onwards (*p* < 0.001, respectively). This age-related trend remained consistent across MS subtypes (Supplementary Figure 3B). Among the DMT-treated population, female and male pwMS received MRIs at similar frequency (Figure 3F). Among the DMT-untreated population of all MS types, female pwMS received more MRIs than male pwMS (*p* < 0.001). A similar pattern was observed across all MS types (Supplementary Figure 3D).

## 4 Discussion

This real-world data analysis, based on claims data from pwMS insured through German SHIs between 2017 and 2022, aimed to assess the proportion of DMT-untreated pwMS according to clinical and sociodemographic characteristics, and the differences in healthcare utilization patterns, between DMT-treated and DMT-untreated pwMS. The analysis of healthcare utilization patterns focused on MRI usage and physiotherapy as key components of MS management in terms of monitoring disease activity and progression, as well as providing symptom-oriented therapy. Additionally, time to first prescription among newly diagnosed pwMS was examined.

Our findings revealed that nearly half of pwMS were not treated with DMTs between 2017 and 2022. This aligns with previous findings from a 2017 German claims data analysis, which reported that 43% of pwMS were not prescribed DMTs (1), and a survey by the German MS Society (2015-2016) indicating that 41.5% of surveyed pwMS did not use DMTs (2). It is important to note that DMT-untreated pwMS may include those who, according to clinical guidelines, are not recommended for DMTs due to absence of relapses, lack of disability progression, or inactive SPMS, i.e., progression without relapses or MRI activity, where there are no licensed treatments available (3). Furthermore, pwMS treated with pulsed therapies such as cladribine or alemtuzumab, may have received treatment cycles outside the observation period, underscoring the proportion of treated pwMS. Similar high shares of DMT-untreated pwMS were found in a United States-based claims data analysis between 2010–2019 and Danish claims data analysis 1995–2015, where 63.6%, and 28.4% of pwMS were not treated with any DMTs in the observation period (4, 5). Discrepancies in DMT treatment rates were largely influenced by type of MS, with pwSPMS and pwPPMS being less frequently DMT -treated compared to pwRRMS, which aligns with results from the Danish claims data analysis were almost twice as many DMT-untreated Danish patients were diagnosed with PPMS than RRMS (5). Previous German claims data analyses from 2017 reported that 81.2% of pwSPMS, 85.8% of pwPPMS, and only 28% of pwRRMS were DMT-untreated (1). Similarly, data from a 2009 Bavarian claims analysis showed that 74.7% of pwSPMS and 80.2% of pwPPMS, but only 41.4% of pwRRMS were not prescribed a DMT (6). While previous German claims data analyses have shown increasing DMT treatment rates over time (2010–2017), this trend was primarily driven by rising DMT treatment rates in the RRMS population (1). In our data, however, we observed a notable increase in DMT treatment rates among pwSPMS and pwPPMS from 2017 to 2022, while DMT treatment rates for pwRRMS remained stable. This shift may in part be attributed to the approval of ocrelizumab as the only DMT for the treatment of pwPPMS in January 2018, and siponimod as the first oral treatment specifically indicated for people with active SPMS in January 2020, expanding treatment options for progressive forms of MS (7, 8). Nevertheless, our data indicate that more DMT treatment options are required for pwPPMS and pwSPMS.

Data from a German prospective cohort study of DMT-naive pwMS showed that the median time from RRMS diagnosis to DMT initiation was 88.0 days (IQR: 52.0–167.0) (9), corresponding to about 3 months, which aligns with our finding that more than half of newly diagnosed pwMS with relapsing disease onset received DMT treatment within the first 6 months of their initial MS coding. In contrast, the time to first prescription for those newly diagnosed with progressive disease onset was significantly longer, with less than a third of pwMS receiving a DMT within 6 months of their initial MS coding.

Our data support previous findings that older pwMS are less frequently treated by DMTs. The Bavarian claims data analysis showed that in 2009, only 32.4% of pwMS under 30 years were untreated compared to 81.5% of those aged 60 and above (6). This may in part be explained by data showing that DMT efficacy tends to decrease with age, potentially contributing to the lower DMT treatment rates observed in older pwMS (10–12). Our analysis revealed that among DMT-treated pwMS, females outnumbered males, while the sex distribution in the untreated population was balanced. This contrasts with a 2016 claims data analysis of pwRRMS with high disease activity (HDA), which found that men (85.1%) received DMT treatment slightly more often than women (81.1%) (13). However, this finding may be skewed to the HDA RRMS subgroup.

Regional differences in DMT treatment rates have not been reported recently, but our analysis surprisingly showed distinct regional differences in rates of untreated pwMS, ranging from 37.5% in eastern Germany to 54.0% in south-western Germany. Unpublished data from the same data source for the same timeframe show that these regional discrepancies in DMT treatment rates are MS-specific and do not pertain to other diseases, e.g., inflammatory bowel disease (Permea platform, proprietary data, 2024). Other studies have previously shown regional discrepancies in the choice of initial MS therapies, most likely due to patient preferences and physician treatment choices (14).

The frequency of physiotherapy sessions varied based on DMT treatment status and MS type. DMT-treated pwMS averaged 20.2 physiotherapy sessions per year, while DMT-untreated pwMS averaged 23.7. A German claims data analysis from 2010 to 2013 found that 55.9% of pwMS received physiotherapy sessions (15). Our results showed that DMT-untreated pwMS received more physiotherapy sessions than DMT-treated pwMS, older pwMS received more physiotherapy sesisions than younger pwMS, and pwSPMS and pwPPMS received more physiotherapy sessions than pwRRMS. The fact that pwMS with a progressive disease course may be characterized by higher disability compared to pwRRMS may in part explain the more frequent use of physiotherapy in progressive MS. In our study, however, disability data were not available to confirm this assumption. Furthermore, the lack of effective DMT treatment options in a large number of progressive pwMS results in increasing importance of symptomatic treatments. Therefore, DMT-untreated pwMS may rely more heavily on symptomatic management to address the functional impairments associated with disease progression, especially in the absence of DMTs, and older pwMS generally require more frequent physiotherapy due to progressive disability (10, 11).

Supporting the assumption that DMT-untreated pwMS would receive fewer MRI scans due to less continuous disease activity monitoring, our findings showed that DMT-treated pwMS averaged around 1.5 MRIs per year, while DMT-untreated pwMS averaged around 0.8 MRIs per year. This is lower than previously reported data in Germany, showing that pwRRMS with HDA had an average three (2.9, SD: 1.9) MRIs per year between 2012 and 2016 (13). This discrepancy is likely due to the increased disease monitoring associated with HDA. Older pwMS received fewer MRIs than younger ones, independent of DMT treatment status. One possible explanation for this finding is that older adults may have more comorbid conditions, which could limit the practicality or necessity of frequent MRI scans (16). Moreover, with increasing age, both relapses and MRI lesion activity tend to become less pronounced, potentially reducing the need for regular imaging (16, 17).

Among DMT-untreated pwMS, females received more MRIs than males. Similarly, we found that among DMT-treated pwMS, females received more physiotherapy than males. The observed sex disparity in physiotherapy and MRI utilization may result from higher health awareness and more frequent healthcare-seeking behavior among women compared to men. A prospective real-world study showed that women with RRMS tend to use more direct medical resources, which could explain their higher rates of symptomatic treatment and disease monitoring (18). Further research is needed to confirm this hypothesis.

### 4.1 Limitations

In addition to the limitations described above, prescriptions recorded in retrospective data sources may not accurately reflect actual medication use, as pwMS might not adhere to prescribed treatments. Additionally, data may be influenced by the quality of healthcare providers' coding. The findings of this study may not be generalizable beyond the study sample, and the sample size may be insufficient for assessing newer or less commonly used compounds. Furthermore, the study sample includes only pwMS insured through statutory health insurance (SHI), excluding those covered by private health insurance, *Freie Heilfürsorge, Beihilfe*, or other insurance plans. In our study, newly diagnosed pwMS were identified by examining their coding history over the past 2 years. However, this method may have led to some misclassification. Patients who were previously diagnosed with MS but took a break from treatment during those 2 years may have been misclassified as newly diagnosed.

## 5 Conclusion

Almost half of pwMS in Germany were not treated with DMTs between 2017 and 2022. PwSPMS and pwPPMS, older pwMS, and those in south-western Germany were more likely to be DMT-untreated, with longer times to treatment initiation among newly diagnosed pwMS with a progressive compared to those with a relapsing disease onset. DMT-untreated and older pwMS received more physiotherapy but less disease monitoring via MRI scans. These results from real-world claims data provide valuable insights to better understand and improve patient care.

## Data Availability

The data analyzed in this study is subject to the following licenses/restrictions. The data are not publicly available due to the private nature of the data. Requests to access these datasets should be directed to benjamin.friedrich@temedica.com.
