# Supplementary material for: Disparities in patient share and characteristics between disease-modifying therapy-treated and -untreated people with multiple sclerosis in Germany: a claims data analysis from 2017 to 2022
Source: Front Neurol. 2025 Apr 15;16:1561810. doi: 10.3389/fneur.2025.1561810 (PMC12037395; doi:10.3389/fneur.2025.1561810)
Supplement: Supplementary file 1 [file Data_Sheet_1.docx]

# Supplementary materials


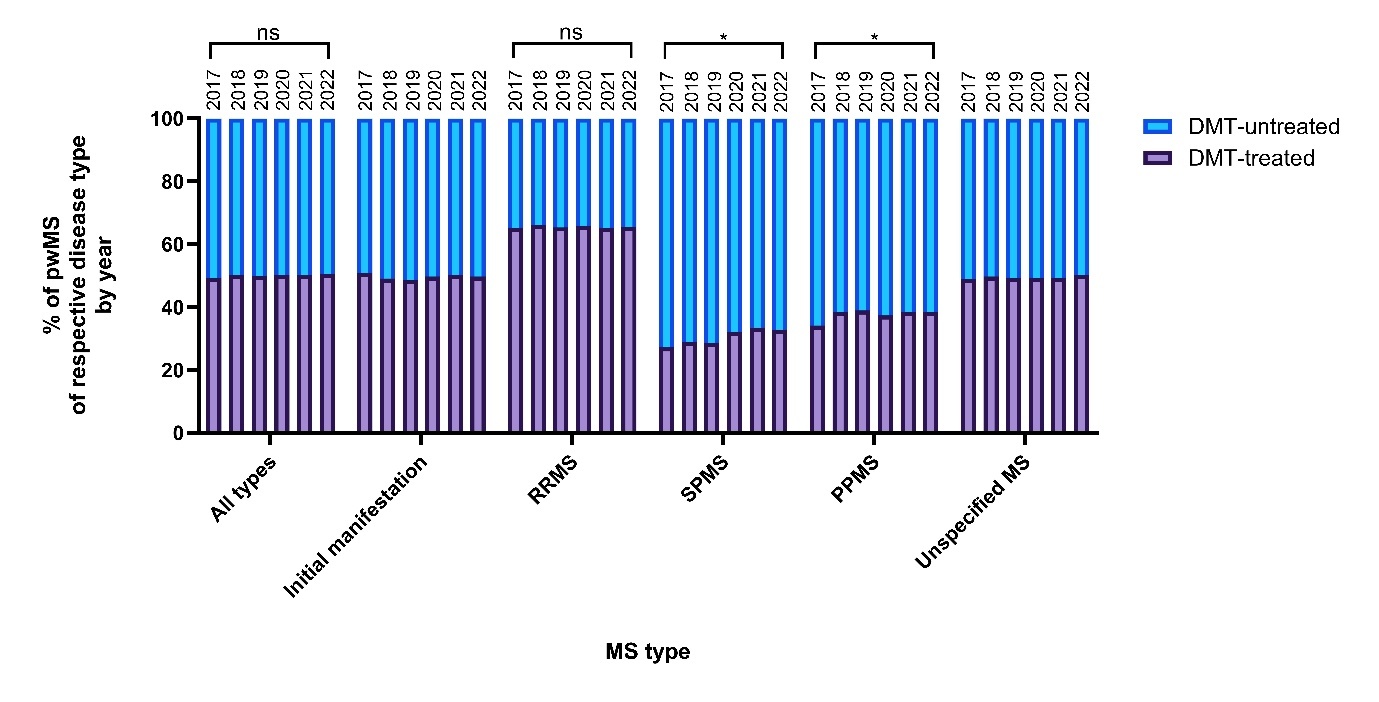


**Supplement Figure 1. Characteristics of DMT-treated and -untreated pwMS by calendar year 2017-2022.** Proportion of DMT-treated and -untreated pwMS, categorized by MS type and calendar year.
Abbreviations: DMT, disease-modifying therapy; MS, multiple sclerosis; pwMS, people with MS.
Differences in proportions of untreated pwMS between 2017 and 2022 were assessed using χ²-tests, with significance levels adjusted for multiple comparisons using the Bonferroni correction (ns: not significant, *significant after Bonferroni correction).


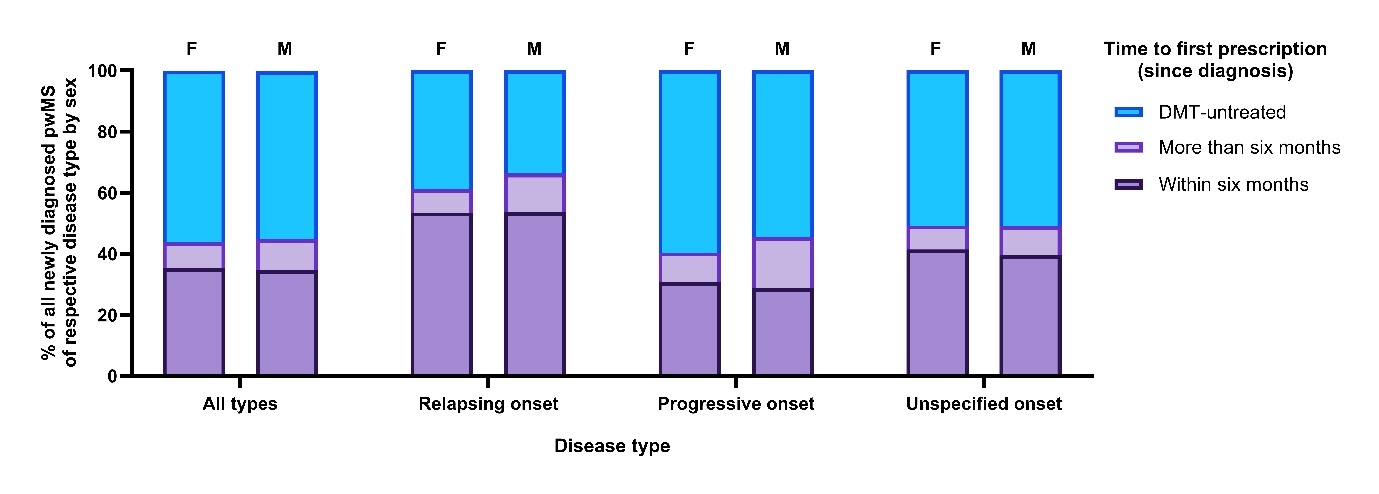


**Supplement Figure 2.** **Time to first prescription since initial MS coding among newly diagnosed pwMS (2017-2022) by MS onset type and sex.** Proportion of newly diagnosed pwMS by MS onset types, categorized by sex who filled a first prescription for a DMT within six months or after more than six months since their initial MS coding or who remained untreated throughout the observation period.
Abbreviations: DMT, disease-modifying therapy; MS, multiple sclerosis; pwMS, people with MS.


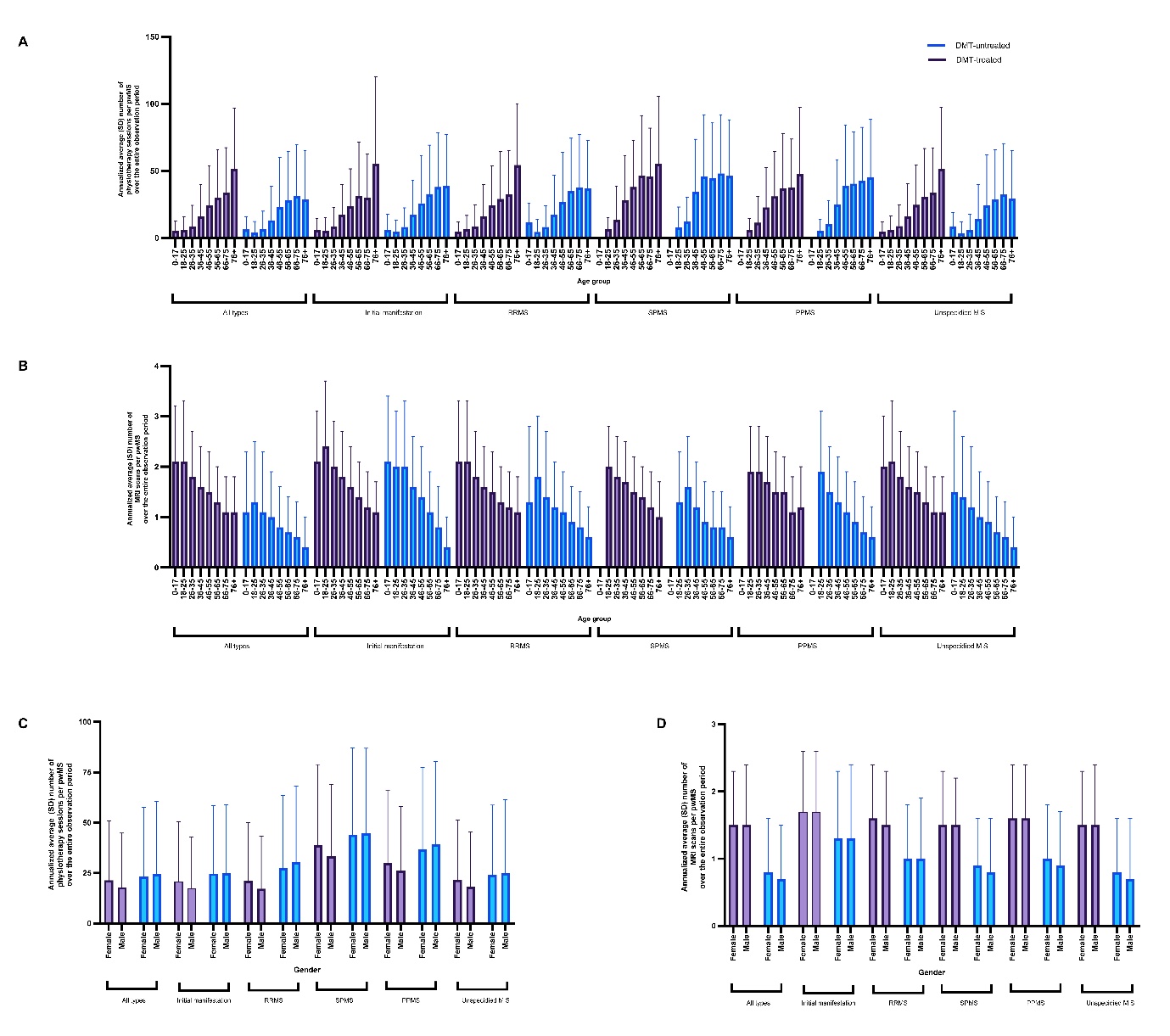


**Supplement Figure 3. Healthcare resource utilization patterns of DMT-treated and -untreated pwMS by MS types, age group and gender (2017-2022). (A & C) Annualized average number (SD) of physiotherapy sessions per pwMS among DMT-treated and -untreated pwMS over the observation period.** **(A)** Categorized by MS type and age group and. **(C)** Categorized by MS type and sex. **(B & D) Annualized average number (SD) of MRI scans per pwMS among DMT-treated and untreated pwMS over the observation period.** **(B)** Categorized by MS type and age group. **(D)** Categorized by MS type and sex.
This analysis includes the total cohort of pwMS, defined as individuals who received an ICD-10-GM code for MS (Initial manifestation of MS, G35.0; RRMS, G35.1-; PPMS, G35.2-; SPMS, G35.3-; and/ or Unspecified MS, G35.9) in at least two quarters in the same calendar year between January 2017 and December 2022.
Abbreviations: DMT, disease-modifying therapy; MRI, magnetic resonance imaging; MS, multiple sclerosis; RRMS, relapsing-remitting MS; SD, standard deviation; SPMS, secondary progressive MS; PPMS, primary progressive MS; pwMS, people with MS.
